# Effects of Blood Flow Restriction Training on Cardiopulmonary Function and Body Composition: A Systematic Review with Meta-Analysis

**DOI:** 10.5114/jhk/204824

**Published:** 2025-05-29

**Authors:** Kun Yang, Chen Soon Chee, Johan bin Abdul Kahar, Tengku Fadilah Tengku Kamalden, Rui Li, Shaowen Qian

**Affiliations:** 1 Department of Sports Studies, Faculty of Educational Studies, Universiti Putra Malaysia, Selangor, Malaysia.; 2 Department of Orthopaedic, Faculty of Medicine and Health Sciences, Universiti Putra Malaysia, Selangor, Malaysia.; 3 National Sports Institute, National Sports Complex, Kuala Lumpur, Malaysia.; 4 Department of Physical Education, Wuhan Sports University, Wuhan, China.

**Keywords:** occlusion training, cardiac function, pulmonary function, muscle hypertrophy, athletes, active participants

## Abstract

The aim of this meta-analysis was to investigate the effects of blood flow restriction training (BFRT) on cardiopulmonary function and body composition of athletes and active participants. Based on the PRISMA guidelines, we searched four international databases for literature up to November 2024, assessed methodological quality using the PEDro scale, and used RevMan 5.4 software for data analysis, publication bias evaluation as well as subgroup analysis. A meta-analysis of forty well-assessed quality studies involving a total of 839 athletes and active participants aged 14–33 years was conducted. The results revealed that BFRT moderately improved both pulmonary function (ES = 0.81–0.88; p < 0.01) and muscle hypertrophy (ES = 0.73–0.74; p < 0.01), while no significant improvement was found for cardiac function (ES = −0.30–0.35; p > 0.05) and anthropometric measures (ES = 0.02–0.04; p > 0.05). Subgroup analyses showed that the moderator variables (training status, age, duration, frequency, training type, and cuff pressure) also had small to large significant effects on pulmonary function and muscle hypertrophy (ES = 0.55–1.74; p < 0.05). In conclusion, BFRT positively affected cardiopulmonary function and body composition in athletes and active participants with significant improvements in pulmonary function and muscle hypertrophy, but not in cardiac function and anthropometric measures. BFRT was more beneficial for improving these physiological metrics when applied to young trained participants with intervention duration of less than six weeks and frequency of fewer than three sessions per week..

## Introduction

With the diversification of scientific training methods, improving cardiopulmonary function and optimizing body composition have become important issues for athletes and active participants. Cardiopulmonary function refers to the body's ability to deliver and utilize oxygen during exercise, with key indicators including the maximum heart rate (HR_max_), the resting heart rate (HR_rest_), cardiac output (CO), stroke volume (SV), maximal oxygen uptake (VO_2max_), and maximal ventilation (VE_max_) (Booher and Smith, 2003; Crisafulli et al., 2011; Park et al., 2010). These indicators not only relate to athletes' sports performance, but also largely determine exercise endurance and cardiovascular health for active individuals (Alvero-Cruz et al., 2020; Zhao et al., 2021). Research has shown that traditional moderate-to-high-intensity endurance training can effectively enhance cardiopulmonary function in athletes. However, for some active populations, particularly those with lower fitness levels, high-intensity training may pose injury risks or even overload the cardiovascular system (Trinity et al., 2012). Consequently, identifying training methods that can improve cardiopulmonary function at lower intensities has become an urgent topic in sports medicine and health sciences.

Blood flow restriction training (BFRT) combines low-intensity exercise with blood flow restriction using special cuffs on the proximal limbs to limit the blood flow. This technique induces high metabolic stress and mechanical tension in muscles under low-intensity conditions, thereby promoting improvements in cardiopulmonary function and muscle adaptation (Sato, 2005). Research has indicated that BFRT can effectively enhance key cardiopulmonary markers, such as VO_2max_ and VE_max_, even under low-intensity conditions, thereby boosting aerobic metabolic capacity and performance in both athletes and active participants (Thompson et al., 2024). Additionally, BFRT has been shown to improve overall cardiovascular health by reducing the resting heart rate and optimizing cardiac output (Renzi et al., 2010).

Besides its impact on cardiopulmonary function, the BFRT's role in regulating body composition is equally noteworthy. Body composition is a critical indicator of physical fitness and health for athletes and active participants and includes measures such as the body mass index (BMI), the body fat percentage (BFP), the muscle cross-sectional area (CSA), and muscle thickness (MT) (Goonasegaran et al., 2012; Roelofs et al., 2015). These metrics not only affect sports performance, but are also closely related to the body metabolism, while optimizing body composition can enhance strength, endurance, and power (Gabbett et al., 2007). Recent studies have found that BFRT can significantly stimulate muscle hypertrophy and improve muscle mass by promoting local metabolic responses and protein synthesis (Geng et al., 2024; Martin et al., 2022). Under low-intensity conditions, BFRT can effectively increase the muscle CSA and MT and play a positive role in reducing the BFP, thus optimizing body composition (Kim et al., 2016; Korkmaz et al., 2022). As such, BFRT is a low-intensity, high-efficiency training approach that not only enhances sports performance and morphology, but also offers a new strategy for fitness maintenance and health promotion in active populations.

Notably, the application of BFRT presents certain health risks and limitations (Brandner et al., 2018). For instance, although BFRT is typically performed at low intensities (20–40% of 1RM or less than 50% of VO_2max_), cardiovascular stress induced by this training modality may pose potential risks for individuals with a high risk of cardiovascular disease (Patterson et al., 2019; Renzi et al., 2010). Therefore, this study exclusively includes healthy individuals as research participants. Furthermore, most previous systematic reviews have primarily focused on BFRT’s effects on muscle strength and hypertrophy, with less emphasis on its effects on cardiopulmonary function and body composition in athletes and active participants (Grønfeldt et al., 2020; Perera et al., 2022). Although the aerobic benefits of BFRT have been demonstrated, its effects on the cardiac function and body composition of athletes and active participants remain controversial. Therefore, this meta-analysis aimed to evaluate the effects of BFRT on cardiopulmonary function and body composition, as well as to examine potential moderators influencing training outcomes. This study could provide scientific support for optimizing BFRT methods and offer safe, efficient training options for populations with varying fitness levels.

## Methods

This meta-analysis followed the PRISMA guidelines (Moher et al., 2009), and registration was completed on inplasy.com (INPLASY202340052U1).

### Search Strategy

We conducted a literature search in the PubMed, Web of Science, EBSCOhost, and Scopus databases for relevant studies published up to November 2024. The detailed search strategy is provided in Table 1. Additionally, we performed supplementary searches via Google Scholar and by examining the reference lists of included studies to address any potential gaps in the database search.

**Table 1:** Search strategies in databases.

Database	Search Strategy	Results
PubMed	(("blood flow restriction training"[Title/Abstract] OR “occlusive training”[Title/Abstract] OR “vascular occlusion”[Title/Abstract] OR “kaatsu”[Title/Abstract] OR “ischemia*”[Title/Abstract]) AND “cardiopulmonary function”[Title/Abstract]) OR “body composition”[Title/Abstract] OR “oxygen uptake”[Title/Abstract] OR “heart rate”[Title/Abstract] OR “muscle hypertrophy”[Title/Abstract]) AND “athlete”[Title/Abstract]) OR “player”[Title/Abstract] OR “active participant”[Title/Abstract]))	839
Web of Science	((TS=(“blood flow restriction training” OR “occlusive training” OR “vascular occlusion” OR “kaatsu” OR “ischemia*”)) AND TS=(“cardiopulmonary function” OR “body composition” OR “oxygen uptake” OR “heart rate” OR “muscle hypertrophy”)) AND TS=(“athlete” OR “player” OR “active participant”)	55
EBSCOhost	AB (“blood flow restriction training” OR “occlusive training” OR “vascular occlusion” OR “kaatsu” OR “ischemia*”) AND TX (“cardiopulmonary function” OR “body composition” OR “oxygen uptake” OR “heart rate” OR “muscle hypertrophy”) AND TX (“athlete” OR “player” OR “active participant”)	44
Scopus	(TITLE-ABS-KEY (“blood flow restriction training” OR “occlusive training” OR “vascular occlusion” OR “kaatsu” OR “ischemia*”) AND TITLE-ABS-KEY (“cardiopulmonary function” OR “body composition” OR “oxygen uptake” OR “heart rate” OR “muscle hypertrophy”) AND TITLE-ABS-KEY (“athlete” OR “player” OR “active participant”))	160

### Study Selection and Data Extraction

Two authors conducted the electronic database searches and imported the literature into EndNote X9 reference management software to automatically remove duplicates. They then performed an initial screening of the titles and abstracts. Following this, the authors reviewed the full texts and selected studies based on the PICOS inclusion criteria. Notably, this review included only studies that investigated the effects of BFRT on cardiopulmonary function and body composition in healthy athletes and active participants. The specific inclusion criteria were: (1) participants were healthy athletes or active individuals (including those with or without regular resistance training experience), with no restrictions on sex, age or the type of sport; (2) the intervention was BFRT or other training combined with blood flow restriction and was not classified as an acute experiment (i.e., not a single-session intervention); (3) a pre-test–post-test, two-group or multi-group experimental design was used; (4) at least one outcome measure related to cardiopulmonary function or body composition (e.g., heart rate, VO_2max_, BFP) was reported; and (5) the study design was a long-term, between-subjects randomized controlled trial. In cases where the two authors disagreed during the screening process, a third author was consulted to reach a consensus.

Data extracted from each study included: (1) authors, the title, and the publication date; (2) participants’ characteristics, including sample size, sex, age, and training status (classified based on whether they had engaged in regular strength or endurance training at least three months before the study); (3) BFRT intervention details (frequency, duration, training protocol, cuff location and pressure, cuff width); and (4) outcome measures. Additionally, Microsoft Excel was used to organize pre- and post-test outcome data for the BFRT and no-BFRT groups.

### Quality Assessment

Two authors assessed the quality of the selected studies using the PEDro scale, which evaluates four main domains: randomization, blinding, comparison, and statistical analysis. The PEDro scale uses a dichotomous scoring system, assigning 1 point for “yes” and 0 points for “no” for each criterion. The total score of each article (excluding eligibility criteria) served as the basis for quality assessment, with a maximum score of 10 (Maher et al., 2003). Quality levels were classified by the total score, with scores ≤ 3 classified as poor, 4–5 as moderate, 6–7 as good, and 8–10 as excellent (Bhogal et al., 2005). In the event of disagreement between the two authors during scoring, a third author was consulted to reach consensus.

### Statistical Analysis

Meta-analysis was conducted using RevMan 5.4 software. In accordance with previous research, the meta-analysis only included data with the same outcome metrics and ≥ 3 studies (Castilla-López et al., 2022). Effect size (ES) was estimated according to the sample size, mean and standard deviation before and after the intervention, and in this study ES was represented by the standardized mean difference (SMD): small (SMD < 0.6), medium (0.6 ≤ SMD ≤ 1.2), and large (SMD > 1.2); additionally, SMD was statistically significant only when *p* < 0.05 (Hopkins et al., 2009). Given the heterogeneity among studies, a random-effects model was applied for pooled analysis. The I^2^ statistic was applied for heterogeneity assessment. Generally, an I^2^ value below 25% indicated low heterogeneity, around 50% suggested moderate heterogeneity, and above 75% indicated high heterogeneity (Higgins and Thompson, 2002). Furthermore, subgroup analyses were performed to investigate sources of heterogeneity and to evaluate a moderator variable's effect, including participants’ training status, age, and BFRT frequency, duration, type, and cuff pressure. Each moderator variable required data from at least three studies and was calculated using the median-split method (Iacobucci et al., 2015). The certainty of evidence for all outcomes was assessed using the GRADEpro tool, with publication bias evaluated through funnel plots available in the software.

## Results

### Study Selection

**Figure 1:**
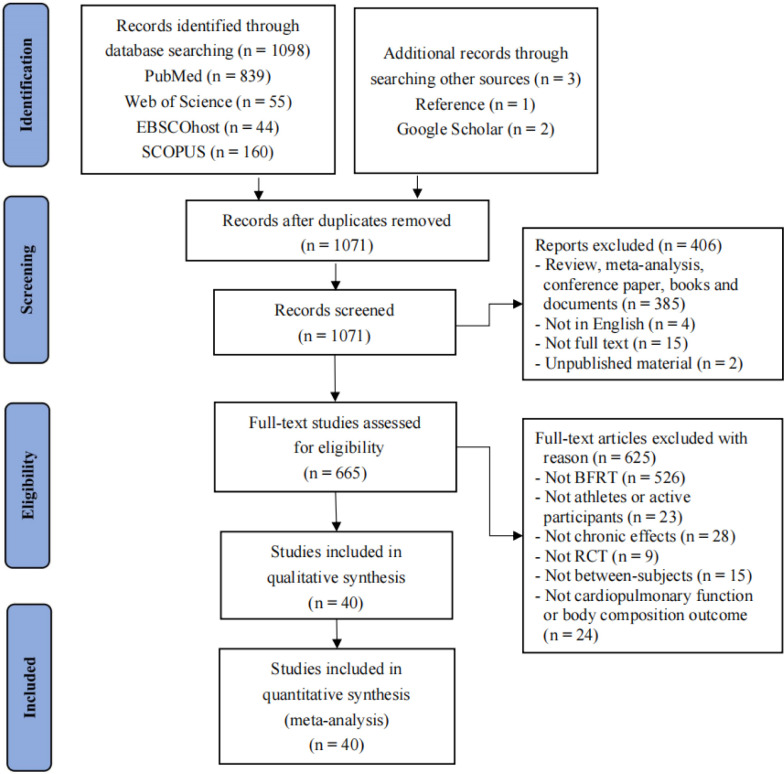
Flow diagram of the search process.

A preliminary search through electronic databases yielded a total of 1,101 records, including three relevant studies identified from reference lists and Google Scholar. After removing 30 duplicates using EndNote X9 reference management software, 1,031 records were further screened based on inclusion criteria, resulting in their exclusion. A total of 40 studies were retained for inclusion in this meta-analysis. The detailed screening and exclusion process is illustrated in Figure 1.

### Study Characteristics

This meta-analysis included 40 randomized controlled trials published between 2002 and 2024, with a total sample size of 839 healthy athletes and active participants. Among them, 580 participants were trained individuals, and 259 were untrained, with a mean age of 22.4 years. All participants were healthy and active, comprising male participants in 29 studies, female participants in 2 studies, and mixed-gender participants in 7 studies. The BFRT intervention characteristics in this study were diverse, with duration ranging from 1.1 to 10 weeks and a frequency of at least two sessions per week. Training protocols included strength exercises (e.g., bench press, squat, knee extension) and endurance exercises (e.g., running, cycling, walking). Specifically, BFRT combined with strength training was classified as of low intensity (< 50% 1RM), while BFRT combined with endurance training ranged from low to high intensity, including low-intensity walking (≤ 6 km/h), moderate-intensity cycling and running (50%–70% heart rate reserve or HR_max_), and high-intensity cycling and running (≥ 80% HR_max_ or maximal speed). The cuffs were placed on the proximal thigh or arm, with pressure ranging from 100 to 240 mmHg and cuff widths between 3 and 18 cm. Specific study characteristics are presented in Table 2.

**Table 2a:** Characteristics of the included studies.

Reference	Population (Type, Sex, N, Age)	Intervention			Outcome
		Duration (Frequency)	Training protocol	Cuff location, Width, Pressure, Application method	
Abe et al., 2005	University track and field athletes, M, EG: 9, CG: 6, 19–22 yr	8 days (14x)	Squat and leg curl, EG/CG: 3 sets × 15 reps/ 20% 1RM	Proximal thighs, 3.3 cm, 160–240 mmHg, Con-BFR	CSA (cm^2^) ↑, MT (cm) ↑, BG (cm) ↑, BMI (kg/m^2^) ↔
Abe et al., 2006	Physically active participants, M, EG: 9, 21.2 ± 2.7 yr, CG: 9, 21.5 ± 2.9 yr	3 weeks (12x)	Walking, EG/CG: 5 sets × 2 min/ 50 m/min	Proximal thighs, 5 cm, 160–230 mmHg, Con-BFR	CSA (cm^2^) ↑
Abe et al., 2010	Physically active participants, M, EG: 9, 23.0 ± 1.7 yr, CG: 10, 23.0 ± 1.7 yr	8 weeks (3x)	Cycling, EG/CG: 1 set × 15–45 min/ 40% VO_2max_	Proximal thighs, 5 cm, 160–210 mmHg, Con-BFR	CSA (cm^2^) ↑, BG (cm) ↑, BMI (kg/m^2^) ↔
Amani et al., 2018	Professional soccer players, M, EG: 10, CG: 9, 23.9 ± 2.3 yr	2 weeks (4x)	Running, EG/CG: 3–4 sets/ 60–70% HRR	Proximal thighs, NR, 140–180 mmHg, NR	VO_2max_ (ml/min/kg) ↑
Amani-Shalamzari et al., 2019	Professional soccer players, M, EG: 6, CG: 6, 23.0 ± 2.0 yr	3 weeks (3x)	SSG, EG/CG: 3 min × 4–8 reps/ 80–100% HR_max_	Proximal thighs, 13 cm, 110–140% SBP, Int-BFR	HR_max_ (bpm) ↑
Amani-Shalamzari et al., 2020	Professional soccer players, M, EG: 6, CG: 6, 23.0 ± 2.0 yr	3 weeks (3x)	SSG, EG/CG: 3 min × 4–8 reps/ 80–100% HR_max_	Proximal thighs, 13 cm, 110–140% SBP, Int-BFR	VO_2max_ (ml/min/kg) ↑
Bagheri et al., 2018	Trained volleyball players, NR, EG: 9, CG: 9, 20–25 yr	8 weeks (3x)	Squat, leg curl and extension, EG/CG: 3–6 sets × 15 reps, 20–30% 1RM	Proximal thighs, NR, 160–240 mmHg, NR	BMI (kg/m^2^) ↔, BF (%) ↔
Behringer et al., 2017	University sprinters, M, EG: 12, 25.6 ± 2.3 yr, CG: 12, 21.7 ± 2.1 yr	6 weeks (2x)	Running, EG/CG: 1 set × 6 reps/ 60–70% Speed_max_	Proximal thighs, 13 cm, Pulled to 75% length, Con-BFR	MT (cm) ↑
Bjørnsen et al., 2019	Elite powerlifters, M&F, EG: 9, 24.0 ± 3.0 yr, CG: 8, 26.0 ± 8.0 yr,	6.5 weeks (5x)	Front squat, EG: 4 sets × 8–30 reps, 30% 1RM, CG: 6–7 sets × 6 reps, 60–85% 1RM	Proximal thighs, 13–14 cm, 120 mmHg, Con-BFR	CSA (cm^2^) ↑, MT (cm) ↑
Castilla-López et al., 2023	Professional soccer players, M, EG: 9, CG: 9, 19.2 ± 1.7 yr	6 weeks (2x)	Back squat, deadlift and barbell, EG: 4 sets × 15 reps, 20–35% 1RM, CG: 4 sets × 8 reps, 70–85% 1RM	Proximal thighs, 7 cm, 160 mmHg, Int-BFR	BG (cm) ↑
Chen et al., 2022	University endurance athletes, M, EG: 10, 21.5 ± 0.8 yr, CG: 10, 21.6 ± 0.8 yr	8 weeks (3x)	Running, EG/CG: 5 sets × 3 min/ 50% HRR	Proximal thighs, 14.2 cm, 154 ± 6 mmHg, Con-BFR	VO_2max_ (ml/min/kg) ↑
Davids et al., 2021	Trained participants, M&F, EG: 11, 23.7 ± 3.1 yr, CG: 10, 24.3 ± 2.9 yr	9 weeks (3x)	Squat, leg press and extension, EG: 4 sets × 15–30 reps, 30–40% 1RM, CG: 4 sets × 8 reps, 75–80% 1RM	Proximal thighs, 10 cm, 60% AOP, Int-BFR	CSA (cm^2^) ↑
de Oliveira et al., 2016	Recreationally active participants, M&F, EG: 10, 26.0 ± 5.0 yr, CG1: 7, 24.0 ± 3.0 yr, CG2: 10, 22.0 ± 7.0 yr	4 weeks (3x)	Cycling, EG/CG1: 2 sets × 5–8 reps, 30% P_max_, CG2: 2 sets × 5–8 reps, 110% P_max_	Proximal thighs, 18 cm, 140–200 mmHg, Int-BFR	VO_2max_ (ml/min/kg) ↑, BMI (kg/m^2^) ↔, BF (%) ↔
Elgammal et al., 2020	University basketball players, M, EG: 12, CG: 12, 22.3 ± 2.4 yr	4 weeks (3x)	Running, EG/CG: 3 sets × 8 reps/ 100% Speed_max_	Proximal thighs, 5 cm, 100–160 mmHg, Int-BFR	VO_2max_ (ml/min/kg) ↑
Giovanna et al., 2022	Endurance athletes, M, EG: 10, 23.9 ± 3.8 yr, CG: 9, 30.2 ± 9.9 yr	2 weeks (3x)	Running, EG/CG: 4 sets × 5 reps/ 100% Speed_max_	Proximal thighs, 11 cm, 45% AOP, Int-BFR	VO_2max_ (ml/min/kg) ↑, VE_max_ (L/min) ↑, HR_max_ (bpm) ↔

**Table 2b:** Characteristics of the included studies.

Reference	Population (Type, Sex, N, Age)	Intervention			Outcome
		Duration (Frequency)	Training protocol	Cuff location, Width, Pressure, Application method	
Held et al., 2020	Elite rowers, M&F, EG: 16, 21.9 ± 3.2 yr, CG: 15, 21.7 ± 3.7 yr	5 weeks (3x)	Rowing, EG/CG: 2 sets × 10 min/ 65% HR_max_	Proximal thighs, 13 cm, Pulled to 75% length, Con-BFR	VO_2max_ (ml/min/kg) ↑
Herda et al., 2024	Trained runners, M&F, EG1: 11, 33.6 ± 10.3 yr, EG2: 11, 30.7 ± 11.2 yr, CG: 11, 33.8 ± 11.2 yr	4 weeks (3x)	Walking, EG1/EG2/CG: 5 sets × 2 min/ 4.83 km/h	Proximal thighs, 10 cm, 80% AOP, Con-BFR	VO_2max_ (ml/min/kg) ↑, HR_max_ (bpm) ↔, BMI (kg/m^2^) ↔, BF (%) ↔
Kim et al., 2016	Physically active participants, M, EG: 11, 23.5 ± 3.4 yr, CG: 10, 21.6 ± 2.5 yr	6 weeks (3x)	Cycling, EG: 20 min, 30% HRR, CG: 20 min, 60–70% HRR	Proximal thighs, 5 cm, 160–180 mmHg, Con-BFR	VO_2max_ (ml/min/kg) ↔, HR_max_ (bpm) ↔, CSA (cm^2^) ↑, BMI (kg/m^2^) ↔, BF (%) ↔
Korkmaz et al., 2022	Professional soccer players, M, EG: 11, 18.4 ± 0.5 yr, CG: 12, 18.4 ± 0.8 yr	6 weeks (2x)	Leg extension, EG: 4 sets × 15–30 reps, 30% 1RM, CG: 4 sets × 12 reps, 80% 1RM	Proximal thighs, 7 cm, 130–150 mmHg, Con-BFR	MT (cm) ↑
Laurentino et al., 2012	Physically active college students, M, EG: 10, 20.0 ± 4.5 yr, CG1: 10, 20.3 ± 4.2 yr, CG2: 9, 23.6 ± 6.0 yr	8 weeks (2x)	Knee extension, EG/CG1: 3–4 sets × 15 reps, 20% 1RM, CG2: 3–4 sets × 8 reps, 80% 1RM	Proximal thighs, 17.5 cm, 80% AOP, Con-BFR	CSA (cm^2^) ↑
Luebbers et al., 2014	University rugby players, M, EG: 17, CG: 14, 20.3 ± 1.1 yr	7 weeks (4x)	Bench press and squat, EG/CG: 4 sets × 20–30 reps/ 20% 1RM	Proximal thighs/ arms, 7.6 cm, Overlap 3 in., Con-BFR	BG (cm) ↑
Manimmanakorn et al., 2013a	University netball players, F, EG: 10, CG: 10, 20.2 ± 3.3 yr	5 weeks (3x)	Knee extension and flexion, EG/CG: 3 sets × 22–36 reps/ 20% 1RM	Proximal thighs, 5 cm, 160–230 mmHg, Con-BFR	VO_2max_ (ml/min/kg) ↑, CSA (cm^2^) ↑
Manimmanakorn et al., 2013b	University netball players, F, EG: 10, CG: 10, 20.2 ± 3.3 yr	5 weeks (3x)	Knee extension and flexion, EG/CG: 3 sets × 22–36 reps/ 20% 1RM	Proximal thighs, 5 cm, 160–230 mmHg, Con-BFR	CSA (cm^2^) ↑
Mitchell et al., 2019	Trained cyclists and triathletes, M, EG: 11, CG: 10, 23.0 ± 5.0 yr	4 weeks (2x)	Cycling, EG/CG: 4–7 sets × 30 s/ 100% Speed_max_	Proximal thighs, 10 cm, 120 mmHg, Int-BFR	VO_2max_ (ml/min/kg) ↑, BMI (kg/m^2^) ↔
Ozaki et al., 2013	Healthy young participants, M, EG: 10, 23.0 ± 0.1 yr, CG: 9, 24.0 ± 1.0 yr	6 weeks (3x)	Bench press, EG: 4 sets × 15–30 reps, 30% 1RM, CG: 3 sets × 10 reps, 75% 1RM	Proximal arms, 3 cm, 100–160 mmHg, Con-BFR	CSA (cm^2^) ↑, BMI (kg/m^2^) ↔
Park et al., 2010	University basketball players, M, EG: 7, 20.1 ± 1.2 yr, CG: 5, 20.8 ± 1.3 yr	2 weeks (12x)	Walking, EG/CG: 5 sets × 3 min/ 4–6 km/h	Proximal thighs, 11 cm, 160–220 mmHg, Int-BFR	VO_2max_ (ml/min/kg) ↑, VE_max_ (L/min) ↑, HR_max_ (bpm) ↔, HR_rest_ (bmp) ↔, SV (ml) ↑, CO (L/min) ↔, BMI (kg/m^2^) ↔, BF (%) ↔
Paton et al., 2017	Physically active participants, M&F, EG: 8, CG: 8, 24.9 ± 6.9 yr	4 weeks (2x)	Running, EG/CG: 2-3 sets × 5–8 reps/ 80% Speed_max_	Proximal thighs, 7.5 cm, Perceived pressure 7/10, Int-BFR	VO_2max_ (ml/min/kg) ↑, VE_max_ (L/min) ↑
Ramis et al., 2020	Physically active participants, M, EG: 15, 23.5 ± 2.8 yr, CG: 13, 24.5 ± 2.6 yr	8 weeks (3x)	Elbow flexion, knee extension, EG: 4 sets × 21–23 reps, 30–40% 1RM, CG: 4 sets × 8 reps, 80% 1RM	Proximal thighs/ arms, 14–16 cm, 100% SBP ± 20 mmHg, Con-BFR	MT (cm) ↑, BF (%) ↔
Sakuraba et al., 2009	University track and field athletes, M, EG: 6, 20.0 ± 0.7 yr, CG: 6, 19.9 ± 0.8 yr	4 weeks (2x)	Knee extension and flexion, EG/CG: 3 sets × 10 reps/ 300°/s	Proximal thighs, NR, 200 mmHg, NR	CSA (cm^2^) ↑

**Table 2c:** Characteristics of the included studies.

Reference	Population (Type, Sex, N, Age)	Intervention			Outcome
		Duration (Frequency)	Training protocol	Cuff location, Width, Pressure, Application method	
Scott et al., 2017	Professional soccer players, M, EG: 10, CG: 8, 19.8 ± 1.5 yr	5 weeks (3x)	Squat, EG/CG: 4 sets × 15–30 reps/ 20–30% 1RM	Proximal thighs, 7.5 cm, Perceived pressure 7/10, Con-BFR	MT (cm) ↑
Takarada et al., 2002	Elite rugby players, M, EG: 12, 25.3 ± 0.8 yr, CG: 12, 26.5 ± 0.7 yr	8 weeks (2x)	Knee extension, EG/CG: 4 sets × 17 reps/ 50% 1RM	Proximal thighs, 3.3 cm, 200 mmHg, Con-BFR	CSA (cm^2^) ↑, BMI (kg/m^2^) ↔
Taylor et al., 2016	Trained cyclists, M, EG: 10, 26.0 ± 5.0 yr, CG: 10, 27.0 ± 7.0 yr	4 weeks (2x)	Cycling, EG/CG: 4–7 sets × 30 s/ 100% Speed_max_	Proximal thighs, 10 cm, 130 mmHg, Int-BFR	VO_2max_ (ml/min/kg) ↑, BMI (kg/m^2^) ↔
Thompson et al., 2024	Recreationally active participants, NR, EG1: 10, EG2: 8, CG: 10, 26.0 ± 2.9 yr	4–6 weeks, (2x)	Walking, EG1/EG2/CG: 5 sets × 3 min/ 5 km/h	Proximal thighs, 11 cm, 100% LOP, Con-BFR	VO_2max_ (ml/min/kg) ↑, VE_max_ (L/min) ↔, HR_max_ (bpm) ↔, BMI (kg/m^2^) ↔
Ugur et al., 2023	Elite canoe players, M, EG: 17, 18.6 ± 0.7 yr, CG: 16, 18.8 ± 1.1 yr	8 weeks (2x)	Leg press, curl and extension, EG/CG: 3–4 sets × 10–15 reps, 30% 1RM	Proximal thighs, 5 cm, 180–230 mmHg, Con-BFR	CSA (cm^2^) ↑, MT (cm) ↑
Wang et al., 2023	University swimmers, M, EG: 8, 19.8 ± 1.2 yr, CG: 8, 20.1 ± 2.0 yr	4 weeks (3x)	Back squat, EG: 4 sets × 15–30 reps, 30% 1RM, CG: 4 sets × 8–12 reps, 70% 1RM	Proximal thighs, 6 cm, 200 mmHg, Int-BFR	HR_rest_ (bpm) ↔, SV (ml) ↔, CO (L/min) ↑, BMI (kg/m^2^) ↔
Yamanaka et al., 2012	University soccer players, M, EG: 16, 19.2 ± 1.8 yr, CG: 16, 19.2 ± 1.8 yr	4 weeks (3x)	Bench press and squat, EG/CG: 4 sets × 20–30 reps/ 20% 1RM	Proximal thighs/ arms, 5 cm, Overlap 2 in., Con-BFR	BG (cm) ↑, BMI (kg/m^2^) ↔
Yang et al., 2022	Professional gymnasts, M&F, EG: 7, 13.9 ± 0.4 yr, CG: 8, 13.8 ± 0.5 yr	10 weeks (2x)	Front and back squat, EG: 3–4 sets × 10–12 reps, 20–30% 1RM, CG: 3–4 sets × 4–5 reps, 60–85% 1RM	Proximal thighs, 7.6 cm, Perceived pressure 7/10, Con-BFR	BG (cm) ↑, BMI (kg/m^2^) ↔
Yasuda et al., 2010	Physically active participants, M, EG: 5, CG: 5, 23–38 yr	2 weeks (12x)	Bench press, EG/CG: 4 sets × 15–30 reps/ 30% 1RM	Proximal arms, 3 cm, 100–160 mmHg, Con-BFR	MT (cm) ↑
Yasuda et al., 2011	Recreationally active participants, M, EG: 10, 23.4 ± 1.3 yr, CG: 10, 25.3 ± 2.9 yr	6 weeks (3x)	Bench press, EG: 4 sets × 15–30 reps, 30% 1RM, CG: 3 sets × 10 reps, 75% 1RM	Proximal arms, 3 cm, 100–160 mmHg, Con-BFR	CSA (cm^2^) ↑
Zhao et al., 2021	Healthy adult participants, M, EG1: 8, 20.0 ± 1.0 yr, EG2: 8, 19.0 ± 1.0 yr, CG: 8, 19.0 ± 1.0 yr	8 weeks (5x)	Elbow flexion and extension, EG1/EG2/CG: 5 sets × 20 reps, 30% 1RM	Proximal arms, NR, 65%–130% SBP, Con-BFR	HR_rest_ (bpm) ↑, SV (ml) ↔, CO (L/min) ↔

Note: M: Male; F: Female; N: Number; NR: Not reported; EG: BFRT; CG: No-BFRT; BFRT: Blood flow restriction training; yr: years; x: sessions/week; SSG: Small sided game; reps: repetitions; 1RM: One repetition maximum; VO_2max_: Maximal oxygen uptake; Speed_max_: Maximal speed; HRR: Heart rate reserve; P_max_: Maximal power; SBP: Systolic blood pressure; AOP: Arterial occlusion pressure; LOP: Lowest occlusion pressure; Con-BFR: Continuous BFR; Int-BFR: Intermittent BFR; VE_max_: Maximal ventilation; HR_max_: Maximal heart rate; HR_rest_: Resting heart rate; SV: Stroke volume; CO: Cardiac output; BMI: Body mass index; BF%: Body fat percentage; CSA: Cross-sectional area; MT: Muscle thickness; BG: Body girth; ↑: Significant improvement; ↔: Non-significant change

### Study Quality Assessment

The included studies were scored using the PEDro scale (Table 3), with results indicating that 40 studies yielded an overall mean score of 6.2, which reflected good methodological quality. Additionally, all included studies demonstrated baseline group similarity, had outcomes available for over 85% of participants, conducted inter-group statistical comparisons, performed intention-to-treat analyses, and reported point measures and/or variability measures. Due to the inability to blind all participants during BFRT interventions, none of the studies employed blinding.

**Table 3:** Physiotherapy Evidence Database (PEDro) scale ratings.

References	1	2	3	4	5	6	7	8	9	10	11	Total Score
Abe et al., 2005	0	1	0	1	0	0	0	1	1	1	1	6
Abe et al., 2006	1	1	0	1	0	0	0	1	1	1	1	6
Abe et al., 2010	1	1	0	1	0	0	0	1	1	1	1	6
Amani et al., 2018	1	1	0	1	0	0	0	1	1	1	1	6
Amani-Shalamzari et al., 2019	1	1	0	1	0	0	0	1	1	1	1	6
Amani-Shalamzari et al., 2020	1	1	0	1	0	0	0	1	1	1	1	6
Bagheri et al., 2018	1	1	0	1	0	0	0	1	1	1	1	6
Behringer et al., 2017	1	1	0	1	0	0	0	1	1	1	1	6
Bjørnsen et al., 2019	1	1	0	1	0	0	0	1	1	1	1	6
Castilla-López et al., 2023	1	1	1	1	0	0	0	1	1	1	1	7
Chen et al., 2022	1	1	0	1	0	0	0	1	1	1	1	6
Davids et al., 2021	1	1	0	1	0	0	0	1	1	1	1	6
de Oliveira et al., 2016	1	1	0	1	0	0	0	1	1	1	1	6
Elgammal et al., 2020	1	1	0	1	0	0	0	1	1	1	1	6
Giovanna et al., 2022	1	1	1	1	0	0	0	1	1	1	1	7
Held et al., 2020	1	1	0	1	0	0	0	1	1	1	1	6
Herda et al., 2024	1	1	1	1	0	0	0	1	1	1	1	7
Kim et al., 2016	1	1	0	1	0	0	0	1	1	1	1	6
Korkmaz et al., 2022	1	1	0	1	0	0	0	1	1	1	1	6
Laurentino et al., 2012	1	1	0	1	0	0	0	1	1	1	1	6
Luebbers et al., 2014	1	1	1	1	0	0	0	1	1	1	1	7
Manimmanakorn et al., 2013a	1	1	0	1	0	0	0	1	1	1	1	6
Manimmanakorn et al., 2013b	1	1	0	1	0	0	0	1	1	1	1	6
Mitchell et al., 2019	1	1	0	1	0	0	0	1	1	1	1	6
Ozaki et al., 2013	1	1	0	1	0	0	1	1	1	1	1	7
Park et al., 2010	1	1	0	1	0	0	0	1	1	1	1	6
Paton et al., 2017	1	1	0	1	0	0	0	1	1	1	1	6
Ramis et al., 2020	1	1	0	1	0	0	1	1	1	1	1	7
Sakuraba et al., 2009	1	1	0	1	0	0	0	1	1	1	1	6
Scott et al., 2017	1	1	0	1	0	0	0	1	1	1	1	6
Takarada et al., 2002	1	1	0	1	0	0	0	1	1	1	1	6
Taylor et al., 2016	1	1	0	1	0	0	0	1	1	1	1	6
Thompson et al., 2024	1	1	0	1	0	0	0	1	1	1	1	6
Ugur et al., 2023	1	1	0	1	0	0	0	1	1	1	1	6
Wang et al., 2023	1	1	0	1	0	0	1	1	1	1	1	7
Yamanaka et al., 2012	1	1	0	1	0	0	0	1	1	1	1	6
Yang et al., 2022	1	1	0	1	0	0	0	1	1	1	1	6
Yasuda et al., 2010	1	1	0	1	0	0	0	1	1	1	1	6
Yasuda et al., 2011	1	1	0	1	0	0	0	1	1	1	1	6
Zhao et al., 2021	1	1	1	1	0	0	0	1	1	1	1	6

Note: Detailed description of each item can be found at *https://pedro.org.au/english/resources/pedroscale/*

### Meta-Analysis Results

**Figure 2:**
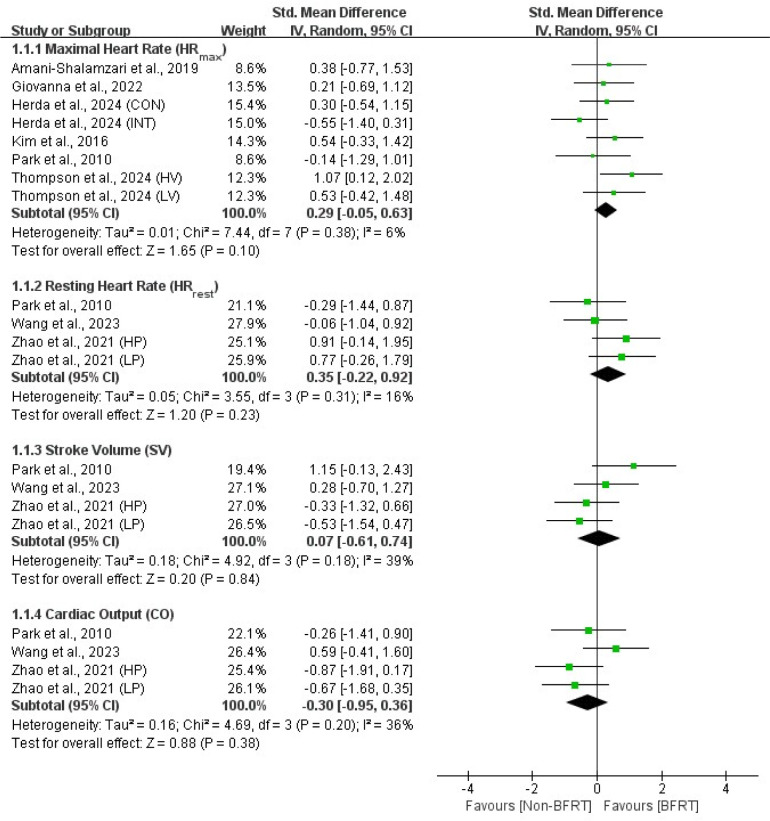
Forest plot of the effects of BFRT versus no-BFRT on cardiac function.

**Figure 3:**
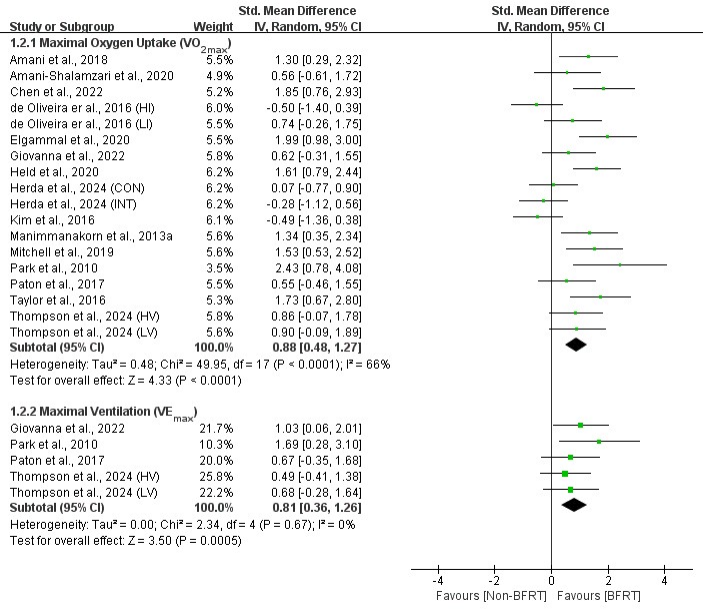
Forest plot of the effects of BFRT versus no-BFRT on pulmonary function.

**Figure 4:**
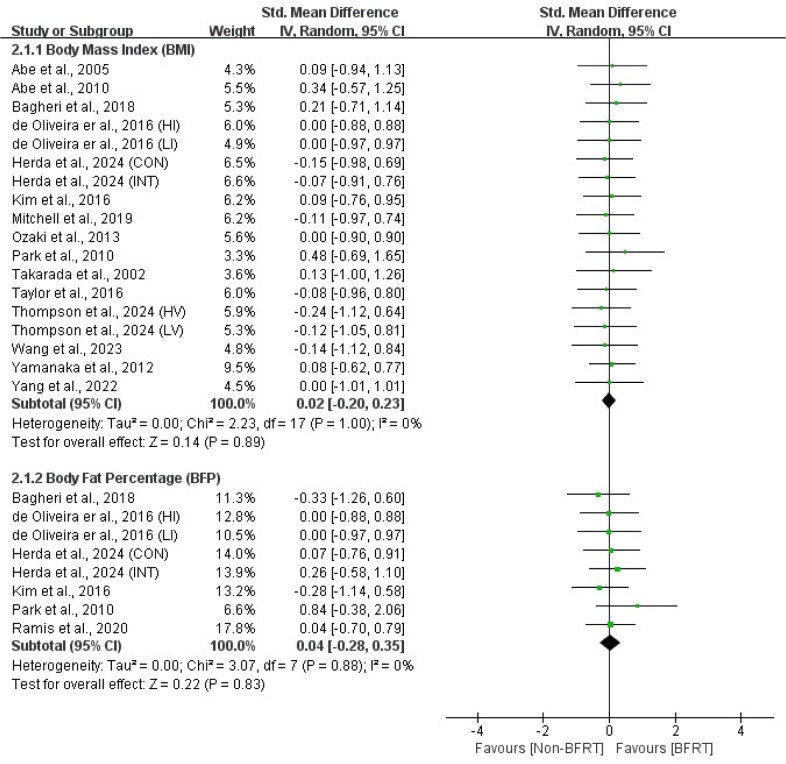
Forest plot of the effects of BFRT versus no-BFRT on anthropometric measures.

**Figure 5:**
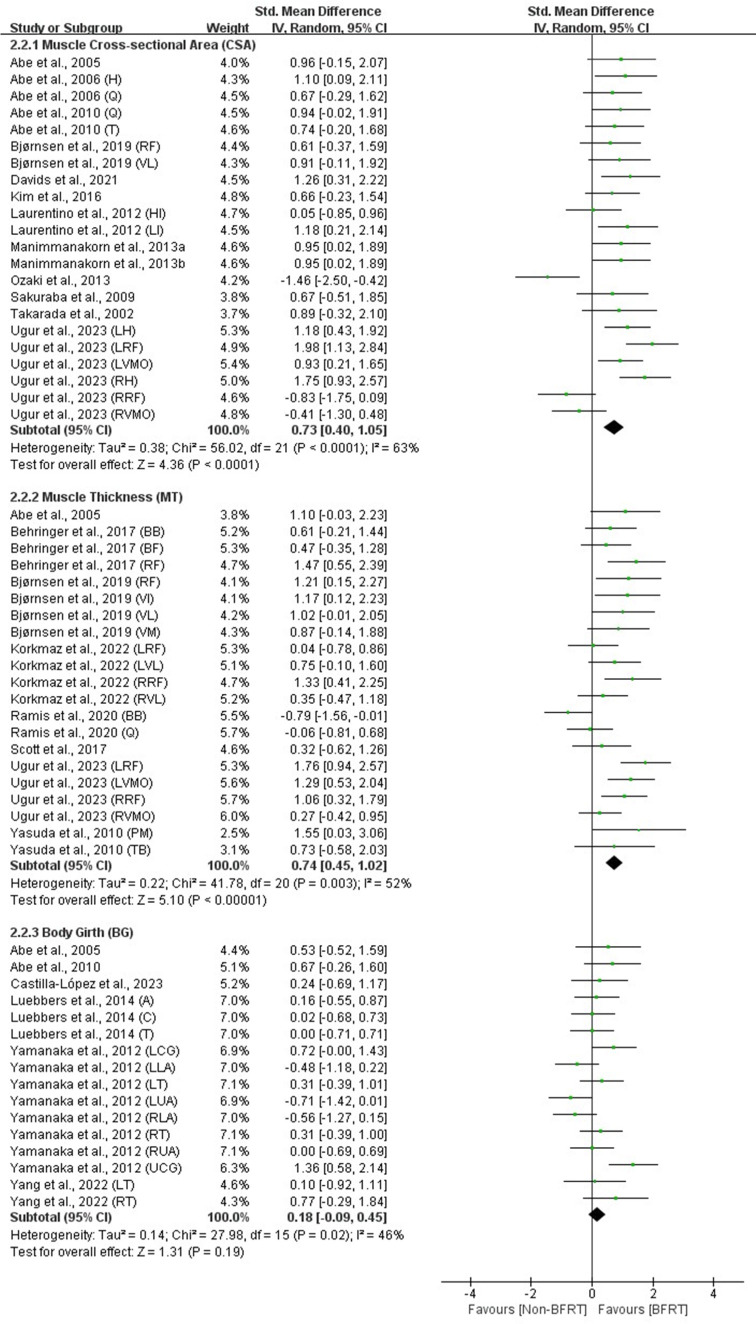
Forest plot of the effects of BFRT versus no-BFRT on muscle hypertrophy.

Outcomes from all included studies on the effects of BFRT on cardiopulmonary function and body composition included measures of cardiac function (HR_max_, HR_rest_, SV, CO), pulmonary function (VO_2max_, VE_max_), anthropometric measures (BMI, BFP), and muscle hypertrophy indicators (muscle CSA, MT, body girth). Detailed results are presented in Figures 2–5.

### Cardiac Function

The meta-analysis of included studies showed no significant differences (*p* > 0.05) between BFRT and no-BFRT groups in HR_max_ (SMD = 0.29; 95% CI = −0.05–0.63), HR_rest_ (SMD = 0.35; 95% CI = −0.22–0.92), SV (SMD = 0.07; 95% CI = −0.61–0.74), and CO (SMD = −0.30; 95% CI = −0.95–0.36). Additionally, the effects were homogeneous across studies (I^2^ = 6%–39%; *p* > 0.05).

### Pulmonary Function

Meta-analysis results indicated that, compared to no-BFRT interventions, BFRT had a moderate and significant effect (*p* < 0.01) on VO_2max_ (SMD = 0.88; 95% CI = 0.48–1.27) and VE_max_ (SMD = 0.81; 95% CI = 0.36–1.26). Moreover, VO_2max_ exhibited moderate heterogeneity (I^2^ = 66%; *p* < 0.01), while VE_max_ showed no heterogeneity (I^2^ = 0%; *p* = 0.67).

### Anthropometric Measures

The meta-analysis results showed no significant differences (*p* > 0.05) between BFRT and non-BFRT regarding the BMI (SMD = 0.02; 95% CI = −0.20–0.23) and the BFP (SMD = 0.04; 95% CI = −0.28–0.35), with no heterogeneity observed (I^2^ = 0%; *p* > 0.05).

### Muscle Hypertrophy

Meta-analysis results indicated that, compared to no-BFRT interventions, BFRT exhibited a moderate and significant effect (*p* < 0.01) on the muscle CSA (SMD = 0.73; 95% CI = 0.40–1.05) and MT (SMD = 0.74; 95% CI = 0.45–1.02). However, there was no significant effect (*p* > 0.05) on the body circumference (SMD = 0.18; 95% CI = −0.09–0.45), with moderate heterogeneity observed across these measures (I^2^ = 46%–63%; *p* < 0.05).

### Subgroup Analyses

Based on the results of the meta-analysis, subgroup analyses were conducted for indicators with significant heterogeneity (I^2^ > 50%; *p* < 0.05), including VO_2max_ in pulmonary function and the muscle CSA and MT in muscle hypertrophy metrics. A total of 18 subgroup analyses were performed, with each moderator factor containing at least three studies, as detailed in Table 4. For the moderator variable related to the study population, results indicated that trained participants under the age of 23 exhibited moderate to large significant effects following BFRT on VO_2max_ (SMD = 1.16–1.74; *p* < 0.01), the muscle CSA (SMD = 0.81–0.86; *p* < 0.05), and MT (SMD = 0.81–0.86; *p* < 0.01) compared to the non-BFRT condition.

**Table 4:** Summary of the effects of moderating variables on pulmonary function and muscle hypertrophy.

Study Characteristics	Subgroups	N	SMD (95% CI)	*p*	Heterogeneity	
					I^2^ (%)	*p*
**Maximal oxygen uptake**						
Training status	Trained	12	1.16 (0.69, 1.64)	< 0.001	62	0.002
	Untrained	6	0.32 (−0.23, 0.86)	0.26	50	0.07
Age	< 23 years	5	1.74 (1.28, 2.20)	< 0.001	0	0.80
	≥ 23 years	13	0.55 (0.15, 0.95)	< 0.001	56	0.007
Duration	< 6 weeks	15	0.91 (0.49, 1.33)	< 0.001	63	< 0.001
	≥ 6 weeks	3	0.71 (−0.61, 2.03)	0.29	82	0.003
Frequency	< 3 sessions/week	5	1.09 (0.65, 1.53)	< 0.001	0	0.45
	≥ 3 sessions/week	13	0.80 (0.28, 1.32)	0.003	73	< 0.001
Training type	Strength training	1	1.34 (0.35, 2.34)	0.008	\	\
	Endurance training	17	0.85 (0.44, 1.27)	< 0.001	67	< 0.001
Cuff pressure	< 160 mmHg	7	0.89 (0.28, 1.50)	0.004	65	0.009
	≥ 160 mmHg	11	0.88 (0.33, 1.42)	0.002	69	< 0.001
**Muscle cross-sectional area**						
Training status	Trained	14	0.86 (0.46, 1.26)	< 0.001	62	0.001
	Untrained	8	0.50 (−0.06, 1.05)	0.08	63	0.008
Age	< 23 years	14	0.81 (0.40, 1.22)	< 0.001	65	< 0.001
	≥ 23 years	8	0.58 (0.02, 1.14)	0.04	61	0.01
Duration	< 6 weeks	6	0.89 (0.48, 1.30)	< 0.001	0	0.99
	≥ 6 weeks	16	0.66 (0.23, 1.10)	0.003	73	< 0.001
Frequency	< 3 sessions/week	10	0.75 (0.18, 1.32)	0.01	75	< 0.001
	≥ 3 sessions/week	12	0.70 (0.33, 1.08)	< 0.001	44	0.05
Training type	Strength training	17	0.70 (0.27, 1.12)	0.001	71	< 0.001
	Endurance training	5	0.81 (0.39, 1.23)	< 0.001	0	0.96
Cuff pressure	< 160 mmHg	3	0.93 (0.37, 1.50)	0.001	0	0.65
	≥ 160 mmHg	19	0.69 (0.32, 1.07)	< 0.001	67	< 0.001
**Muscle thickness**						
Training status	Trained	17	0.86 (0.62, 1.10)	< 0.001	22	0.20
	Untrained	4	0.18 (−0.71, 1.06)	0.69	67	0.03
Age	< 23 years	10	0.81 (0.46, 1.17)	< 0.001	46	0.05
	≥ 23 years	11	0.67 (0.22, 1.12)	0.003	58	0.009
Duration	< 6 weeks	4	0.79 (0.21, 1.37)	0.008	0	0.53
	≥ 6 weeks	17	0.72 (0.40, 1.05)	< 0.001	59	< 0.001
Frequency	< 3 sessions/week	11	0.84 (0.51, 1.17)	< 0.001	45	0.05
	≥ 3 sessions/week	10	0.62 (0.13, 1.10)	0.01	57	0.01
Training type	Strength training	18	0.72 (0.40, 1.05)	< 0.001	56	0.002
	Endurance training	3	0.82 (0.23, 1.40)	0.006	30	0.24
Cuff pressure	< 160 mmHg	16	0.61 (0.29, 0.93)	< 0.001	48	0.02
	≥ 160 mmHg	5	1.07 (0.55, 1.58)	< 0.001	51	0.08

Note: N: Number of trials; SMD: Standardized mean difference; CI: Confidence interval

Regarding the moderator variables related to training interventions, BFRT demonstrated moderate significant effects on VO_2max_, the muscle CSA, and MT compared to the non-BFRT condition when the intervention duration was less than six weeks (SMD = 0.79–0.91; *p* < 0.01), frequency was less than three times per week (SMD = 0.75–1.09; *p* < 0.05), and when involving endurance training (SMD = 0.81–0.85; *p* < 0.01). Additionally, when cuff pressure was less than 160 mmHg, BFRT showed superior improvements in VO_2max_ and the muscle CSA, while effects on MT were more pronounced at cuff pressures ≥ 160 mmHg.

### Certainty of Evidence

This study utilized the GRADE approach to assess the certainty of evidence for 11 outcomes. Funnel plots for all outcomes were symmetrical, indicating no significant publication bias. Furthermore, the analysis revealed that the certainty of evidence for the BMI and body girth was rated as moderate, while the certainty of evidence for all other outcomes was rated as low, as detailed in Table 5.

**Table 5:** GRADE assessment of the results.

Certainty assessment						
Studies, Participants (EG/CG)	Risk of bias	Inconsistency	Indirectness	Imprecision	Publication bias	Certainty of evidence
Maximal heart rate 8 RCTs, 74/72	Serious^a^	Not serious	Not serious	Serious^c^	None	⊕⊕○○ Low
Resting heart rate 4 RCTs, 31/29	Serious^a^	Not serious	Not serious	Serious^c^	None	⊕⊕○○ Low
Stroke volume 4 RCTs, 31/29	Serious^a^	Not serious	Not serious	Serious^c^	None	⊕⊕○○ Low
Cardiac output 4 RCTs, 31/29	Serious^a^	Not serious	Not serious	Serious^c^	None	⊕⊕○○ Low
Maximal oxygen uptake 18 RCTs, 181/173	Serious^a^	Serious^b^	Not serious	Not serious	None	⊕⊕○○ Low
Maximal ventilation 5 RCTs, 43/42	Serious^a^	Not serious	Not serious	Serious^c^	None	⊕⊕○○ Low
Body mass index 18 RCTs, 173/166	Serious^a^	Not serious	Not serious	Not serious	None	⊕⊕⊕○ Moderate
Body fat percentage 8 RCTs, 84/76	Serious^a^	Not serious	Not serious	Serious^c^	None	⊕⊕○○ Low
Muscle CSA 22 RCTs, 235/224	Serious^a^	Serious^b^	Not serious	Not serious	None	⊕⊕○○ Low
Muscle thickness 21 RCTs, 243/230	Serious^a^	Serious^b^	Not serious	Not serious	None	⊕⊕○○ Low
Body girth 16 RCTs, 220/211	Serious^a^	Not serious	Not serious	Not serious	None	⊕⊕⊕○ Moderate

Note: EG: BFRT; CG: No-BFRT; RCTs: Randomized controlled trials; CSA: Cross-sectional area; a: Lack of blinding during study execution; b: I-square greater than 50%; c: Insufficient sample size

## Discussion

We conducted a meta-analysis of 40 studies with good quality assessments, encompassing a total sample size of 839 healthy athletes and active participants aged 14 to 33 years. The results indicated that compared to no-BFRT, BFRT interventions led to moderate improvements in pulmonary function (VO_2max_ and VE_max_) and muscle hypertrophy (muscle CSA and MT) (ES = 0.73–0.88). However, there were no significant improvements (*p* > 0.05) in cardiac function (HR_max_, HR_rest_, SV, and CO), anthropometric measures (BMI and BFP), and body girth. Furthermore, subgroup analyses revealed that, compared to the non-BFRT condition, trained participants under the age of 23 demonstrated moderate improvements in VO_2max_, the muscle CSA, and MT when undergoing endurance training interventions lasting less than six weeks and occurring less than three times per week.

Cardiac function refers to the heart's ability to maintain a stable blood flow within the circulatory system to meet the body's energy supply demands at varying intensities of exercise (Green et al., 1990). The findings of this study showed no significant improvements in cardiac function indicators with BFRT compared to no-BFRT (*p* > 0.05). Existing meta-analyses have also indicated no significant differences in the HR_rest_ between BFRT and no-BFRT in general populations and older adults (Wong et al., 2022; Zhang et al., 2022). However, BFRT has been shown to enhance the heart rate and cardiac output in young individuals in response to acute cardiovascular demands (Lemos et al., 2022). It is noteworthy that improvements in cardiac function may be related to acute compensatory changes in distal venous blood flow circulation due to cuff pressure (Fernandes et al., 2025; Pope et al., 2013). Specifically, during short-term BFRT, restricted venous return and increased vascular resistance can lead to decreased SV and an increased heart rate, thereby enhancing CO to maintain energy supply during exercise (Renzi et al., 2010). Therefore, long-term BFRT did not demonstrate significant positive effects on the improvement of cardiac function among athletes and active individuals. Additionally, the intervention protocols of BFRT may influence cardiac function indicators, and the existing literature on HR_rest_, SV, and CO is limited (only three studies available), thus necessitating cautious interpretation of the findings related to cardiac function.

Pulmonary function reflects the ability of the lungs to inhale, exchange gases, and exhale air at different activity levels (Inbar et al., 1993). The results indicated that BFRT had a moderate significant effect on pulmonary function variables compared to no-BFRT (ES = 0.81–0.88). Previous meta-analyses have also shown that BFRT significantly improves VO_2max_ in healthy young individuals compared to no-BFRT (Formiga et al., 2020). However, the meta-analysis by Castilla-López et al. (2022) found that while aerobic capacity improved in athletes following BFRT, the intergroup differences compared to no-BFRT were not statistically significant (ES = 1.02; *p* = 0.064). In fact, the enhancement of aerobic capacity in pulmonary function may be attributed to dual adaptations at both central and peripheral levels, which accelerate physiological metabolism and synthetic responses, such as increased erythropoietin secretion in hypoxic environments and after exercise, thereby enhancing red blood cell and hemoglobin levels and improving oxygen delivery capacity and adaptability (Keramidas et al., 2012; Koistinen et al., 2000). During BFRT, ischemia and hypoxia modulate vascular endothelial growth factor and endothelium-dependent vasodilation, improving the efficiency of oxygen and carbon dioxide exchange as well as exercise endurance (Formiga et al., 2020). Therefore, BFRT demonstrates a more positive effect on pulmonary function among athletes and active individuals. Subgroup analyses showed that younger, trained participants experienced significant increases in VO_2max_ (ES = 0.85–1.74) following endurance training twice a week for less than six weeks. Previous meta-analyses have indicated that when cuff pressure is ≥ 130 mmHg, combining two to four weeks of aerobic exercise with BFRT leads to more pronounced improvements in aerobic performance in healthy young individuals (Bennett and Slattery, 2019; Yang et al., 2022). Notably, compared to strength training, endurance training (such as cycling and running) has a more pronounced effect on pulmonary function, primarily due to its long-term adaptive stimulation of the aerobic metabolism. Studies have shown that endurance training can promote capillary formation and mitochondrial biogenesis in skeletal muscles, thereby enhancing oxygen utilization efficiency (Holloszy and Coyle, 1984). Additionally, this may be attributed to the higher physiological adaptability and neural regulation capacity of young adolescents, as well as their lower metabolic accumulation (Radnor et al., 2018). Low-frequency, low-cycle BFRT may be more beneficial in activating aerobic metabolic pathways in the muscles of trained young individuals, promoting angiogenesis and enhancing oxygen utilization, ultimately improving the aerobic metabolism and maximum ventilation capacity (Formiga et al., 2020; Yang et al., 2022). The findings of this study suggest that low-frequency, low-cycle endurance training has more significant benefits for pulmonary function in trained young populations.

Anthropometric measures reflect body composition or proportions and are primarily used to assess the overall physical health status (Bhattacharya et al., 2019). The results of this study revealed that BFRT did not lead to significant improvements in anthropometric measures compared to no-BFRT (*p* > 0.05). However, a meta-analysis by Sun (2022) found that BFRT significantly reduced the BMI in obese adults (*p* = 0.02), although it had no significant effect on the BFP (*p* = 0.10). From a physiological perspective, the increased local metabolic stress experienced by obese adults during BFRT induces higher levels of lactate accumulation, which stimulates the release of growth hormone, promoting muscle synthesis and energy expenditure, thereby contributing to a reduction in the BMI (Karabulut and Garcia, 2017). It is important to note that significant reductions in the BFP primarily depend on lipolysis, and BFRT is less effective than traditional high-intensity training in initiating fat metabolism pathways, making it difficult to lower the body fat percentage through fat consumption alone (de Oliveira et al., 2016). For individuals of normal weight, the effects of metabolic stress from BFRT on the BMI and the BFP are relatively weak due to the stability of muscle-to-fat ratios, as well as lower basal metabolic rates and body fat levels (Kim et al., 2016). Therefore, the improvement effects of BFRT on anthropometric measures in athletes and active individuals are not significant.

Muscle hypertrophy indicators primarily assess changes in muscle size, shape, and growth, serving as measures of muscle quality (Buckner et al., 2016). The results of this study demonstrated that BFRT had a moderate significant impact on the muscle CSA and MT compared to no-BFRT (ES = 0.73–0.74), while it showed no significant effect on the body girth (ES = 0.18, *p* = 0.19). Previous meta-analyses have also indicated that BFRT yields greater improvements in muscle hypertrophy in trained individuals compared to high-intensity resistance training (HI-RT) (Geng et al., 2024). However, a meta-analysis by Lixandrao et al. (2018) found no significant difference in muscle quality improvement between BFRT and HI-RT for the general population and older adults. It is noteworthy that differences in sample size, study populations, and training status may significantly influence the analytical outcomes of these two meta-analyses. Muscle hypertrophy can be attributed to the activation of muscle growth pathways (such as mTOR and calcium neuron signaling pathways), leading to changes in muscle fibers and metabolic demands (Bodine et al., 2001; Fry et al., 2010). BFRT increases metabolic stress in muscles by restricting the blood flow, inducing the accumulation of metabolites such as lactate, causing moderate muscle damage, and promoting protein synthesis, thereby stimulating repair and remodeling mechanisms to enhance muscle hypertrophy (Fujita et al., 2007). Therefore, BFRT shows a more positive effect on muscle hypertrophy indicators in athletes and active individuals. Subgroup analyses indicated that younger, trained participants exhibited significant improvements in muscle hypertrophy after engaging in endurance training twice a week for less than six weeks (ES = 0.75–0.89). Previous meta-analyses have suggested that variations in age, duration, and frequency of training do not significantly affect the hypertrophy outcomes of BFRT in untrained individuals (Geng et al., 2024). Importantly, training status is considered a key factor influencing muscle hypertrophy outcomes, and the occlusion pressure does not serve as a determining factor for hypertrophy effects between BFRT and HI-RT (Geng et al., 2024; Lixandrao et al., 2018). Trained individuals possess higher muscle activation, metabolic stress tolerance, muscle repair capabilities, and cardiovascular adaptations. In contrast, active individuals without regular resistance training experience may have more difficulty in adapting to the high metabolic stress induced by blood flow restriction. Consequently, trained individuals exhibit significantly greater hypertrophy effects following BFRT compared to untrained individuals. This may be because younger trained individuals are more likely to activate cellular signaling pathways such as mTOR and ERK1/2 in the short term, enhancing the expression of growth factors and promoting muscle growth (Gundermann et al., 2012). Therefore, low-frequency and low-cycle endurance training has a more positive impact on muscle hypertrophy indicators in young trained individuals.

This meta-analysis has several limitations. Firstly, the data on cardiac function (such as HR_rest_, SV, and CO) included in the studies were limited, comprising only three studies, which may restrict the reliability of the analysis results for these metrics. Secondly, this study did not account for gender differences. Given that the study population primarily consisted of male participants, there are insufficient female data to conduct subgroup analyses. Therefore, future high-quality research is needed to further validate and refine the predictive results of this analysis, providing a more comprehensive theoretical basis for BFRT.

## Conclusions

This meta-analysis confirms the positive effects of BFRT on cardiovascular and pulmonary function as well as body composition in athletes and active participants. Specifically, the evidence indicates that BFRT significantly improves pulmonary function and muscle hypertrophy, while showing no significant effects on cardiac function and anthropometric measures. However, data regarding the impact of BFRT on cardiovascular variables such as HR_rest_, SV, CO, and VE_max_ remain limited, with a low certainty of evidence, necessitating further research for validation. Subgroup analysis results indicate that BFRT is more beneficial for improving these physiological metrics when applied to young trained participants with the intervention duration of less than six weeks and a frequency of fewer than three sessions per week.
